# Proceedings of the North Carolina Dental Society, Winston, N. C., May 24-26, 1892

**Published:** 1892-10

**Authors:** 


AMERICAN JOURNAL

—OF—

DENTAL SCIENCE.

Vol. xxvi. Baltimore, Octobber, 1892. No. 6.

ARTICLE I.

### PROCEEDINGS OF THE NORTH CAROLINA DENTAL SOCIETY, WINSTON, N. C., MAY 24-26, 1892.

### AFTERNOON SESSION, MAY 24.

The Minutes of last meeting read and approved.

The essay of Dr. J. H. Durham, of Washington, on "The North Carolina State Dental Society," was received with hearty applause.

Dr. H. C. Herring, of Concord, introduced Dr. M. H. Cryer, of Philadelphia, who spoke as follows:

"Mr. President and members of the North Carolina Dental Association: I feel proud in being invited by your President to meet you here. From what I see we shall have, I think, a very pleasant time, and it will be entertaining and instructive to all of us. It gives me great pleasure to meet one member of my old graduating class of 1876; it is the first that I have seen of him since that time, and I was glad to have him tap me on the shoulder and say, 'Hello, Matt, how are you?' There are also several members here whom I have met before in Philadelphia, and it gives me great pleasure to meet them again.

I have been invited to perform any surgical operation that might be presented, and I only hope that we may have a number of cases which will be of interest to this Association. I have seen two patients, one of whom has had a cancer removed from the left side of his nose. He is an elderly person and from the character of the surrounding tissue I am unwilling to perform a plastic operation as I was in hopes of doing; I fear it might leave the man in a worse condition than he is. The only thing remaining to be done, then, is to perform a purely mechanical operation by covering the opening made by the removal of the cancer with a piece of celluloid or Vulcanite and have it painted the color of the face; this, I think, can be held in position and will make the patient presentable. An occulist is now at work adjusting a pair of spectacle frames and after they are ready we will commence with a wax model, to cover or fill in this opening as near as it can be made to the other side of the nose. After that it will be transferred to vulcanite. There is another case, of which I only had a glance, and that was a cleft palate. As far as I can see, it will be a case for "mechanical work."

Moved and seconded that the visiting dentists be accorded the privileges of the floor.

Committee on Dental Education and Literature responded with two papers, one by Dr. J. H. Loudan and another by Dr. I. N. Carr.

Dr. Durham said: I wish to express my hearty approval of the papers of both Dr. Loudan and Dr. Carr. I think Dr. Loudan has made some very valuable practical suggestions, and I think Dr. Carr's paper is as good a paper upon that subject as I ever heard. Dental education is really the life blood of our society and of dentistry, and that is the only way you can elevate the profession morally and intellectually.

Dr. Morgan, of Nashville, Tenn., said: I wish to commend the gentlemen for the bold manner in which they speak their sentiments and belief; the young man who read the last paper (Dr. Loudan) deserves particular credit. In the first paper I was very glad to hear the gentleman qualify what Dr. Wood had said with reference to medical schools. I understood him to say it applied to the medical colleges. I differ, however, with the gentleman and with Dr. Wood; I think Dr. Wood's language is entirely too strong and is an insult to his diploma. If the gentleman who read the paper believes that the schools in this country are not endeavoring to do their duty as much as the North Carolina Dental Association and the North Carolina Dental Board, I think he is mistaken in that. Dental education is a subject that will agitate the minds of the profession as long as we claim to be a profession. There are dentists to be educated, and the demands of the dentists how best to educate them will be an unsolved problem at the end of time. I notice both gentlemen commended very highly the plan of pupilage previous to the entrance into college. I believe the experience of college men will bear me out in the assertion that unless you place your pupils in the office of such men as Griffith and Hunter and Turner, or other men of that character, their office pupilage will be a detriment to them instead of an advantage. The experience of college men on this subject has been that those men who have earned their way through college, who came there with an earnest desire to acquire a knowledge of dentistry, that they may be able to place themselves alongside of the professional men of their State, with a just conception of what the word "professional" means, are the students who do best whether they come from a dental office or from behind plow handles, and my observation has been that these are best and not those who come from an office, as a rule. The pupil from the average office has as much to unlearn as he has to learn, and it takes just about as long to unlearn it and be set right after he comes to college as it does to learn it properly in the first instance.

Dr. B. Holly Smith, of Baltimore, said: The subject of dental education has grown to be an amusing one to me. It seems to be a popular plan in our dental societies to invite an essayist to deliver an oration upon the weakness and faults of dental schools and then to give the teacher an opportunity to reply and exonerate the institution. I do not stand here in defence of the dental college. I commend the sentiments of the paper which the first essayist read in regard to the cultivation of character and the hints and suggestions to young men, but I must take on a slight exception to it, and repeat the statement which I made before in regard to the forcing of colleges to extend the course. So far as the extension of the course is concerned, it has been more the result of a recognition on the part of the faculties of the various colleges than sentiment in the profession. Without any demand on the part of associations, colleges are eager and anxious to take such steps as would answer that demand, so it is not a matter between colleges and the profession. Gentlemen, we are all graduates, or ought to be, of some dental college and we remember with pleasure the first early trainings in our Alma Mater; let us not go away from them; it would be a sad day if we were to begin to complain of their educational influences. What we want to do is to uphold the work and encourage rather than to find fault.

I was particularly pleased with the second essayist's paper. It is a source of considerable gratification to see a young man who worked with me and under me, come forward with so many valuable suggestions. As for your dental societies it strikes me that in this line of dental education there can be more done by encouraging a man. After we give you men from a dental college, we have done what we could; then let him go to work if it be nothing more than to make a scrap book, but as for giving him professional standing we cannot do that.

In regard to the comparison which was made between

European and American education, right along that line I saw a little discussion between Pres. Elliott and a member of the New York School Board in regard to the comparison between education in Europe and America. There always will be a difference of opinion as to the merits and demerits in comparing one system with another. So far as my experience goes I will say, not because I want to boast of our institutions, that there can be no doubt of the supremacy of the teachings and of the results in American schools of dentistry. I believe that in medicine we do not see the results quite, that they do in Europe. The German experimentors have developed and brought forward instruments that our American students have been slow to learn the use of even, but I do not think that is the way in dentistry so far as I am able to judge. In regard to the subject of dental education, I would reiterate let us try so far as is practicable to encourage the schools to do what they would like to do—they would like to do all that is possible on the line of dental education. Let us have unanimity and uniformity in any demands. Don't demand here in North Carolina that they shall attend three or four years and then go down to Texas and say it is an outrage, and that the colleges are trying to get more money out of the pupils by one more year. Let us have uniformity of sentiment, and if there is any difference let us come together and compare notes, and through our National Association let us harmonize any conflicting elements.

Dr. Carr: I hardly feel capable of replying to the gentleman that has just discussed some of the statements that I made in the paper that I read before you. I would like to say in the outset that it was far from my intention to say anything derogatory to any college in the United States of a reputable character. So far as the college of Nashville, Tenn., is concerned, which our good brother represents, we all know that there is none better. I quoted Prof. H. C. Wood—quoted his language, I think, ver batim—and it was not that I think colleges are not necessary to educate the young men. But my idea was that young men that are taken into college without any preliminary education will certainly require a longer term of study to acquire the necessary knowledge to practice dentistry successfully, when on the contrary, after he takes a year or two of pupilage under some reputable dentist, I think he is better prepared to enter college and will be a great deal more credit to the profession than if he started into college at the first. None know better than myself how much good work these grand institutions of ours have done, and I am proud of it. There is nothing that I would not be perfectly willing to do to help them on in their grand undertaking to educate the young men of this country in dentistry, and I hope the gentleman will not think that my paper was intended in any particular as the slightest reflection upon any of the colleges of this country.

Dr. C. A. Rominger: I want to say that that is the bed rock upon which we ought to build—this subject of dental education—and doubtless all of us here feel the necessity of early education, and many of us have gone to work when we were unequipped, and now as true dentists, and desirous of elevating the profession and holding out before the youth of this country any ideal course, I say, with that idea before us, we ought to lay broad our foundation. We ought to have our idea clear cut that you may offer the very best advantages to those who seek to enter our profession, that we may seek to elevate not only the mechanical work of the profession but the standing of our profession in the estimation of the public of our State. I feel proud when I look around upon the members of our profession, because of the scope of their information, because of the skill with which they execute their work. Those of us who were at the clinics feel proud of the hands and skill of those who operate at the chairs here in our association; but when we go back to our homes in the months and years to follow, we hear much that comes into our offices from those men who do not come to our

Associations. A number of men are practicing dentistry over the land who would not come to our meetings. They shut themselves off from all improvement, and there are some young men coming into the profession just that way and shutting themselves off from the facilities which we would extend to them if they would allow us. So I say we want to lay a broad foundation and hold out an inviting hand to these and say "come in and learn; be benefactors to the human race, and not those who simply are seeking to use the very best means they can get for their own selfish purposes." We know too well how much the public is damaged in that way.

Another thing: There ought to be no conflict between our societies and our colleges; we are all laboring for the same great end. We ask all the dentists of North Carolina to come here and help us and be helped; and the colleges say to the young men, "come here and take our course; we offer you the best instruction that can be given in this land." And so we ought to lay broad the foundation and encourage the young men who are coming into the profession, so that they may reflect honor upon the profession and that they may be an honor to themselves and a blessing to many. We do not demand sufficient literary education at times, and men are brought into the profession with such a meagre education in literature, that when they come to discuss the questions of the day, their opportunities have been so limited that they are incompetent and do not elevate the profession as they should. Well has Gray said:

> "Full many a flower is born to blush unseen,
> And waste its sweetness on the desert air."

Young men of great powers of intellect, but whose powers are lying dormant, never having been developed and brought out and polished and made fit to adorn him as the jewel does the beautiful maiden, so when they come to you and ask what are the requirements of a dental education, urge them to go to school that they may fit themselves and bring out these latent powers and be really useful men, men to be felt, men to execute well the work of the profession which they chose. Let us encourage this subject of dental education and make it broad and help to elevate the standard and give high aspirations and give them the benefit of the instructions and experience which we have gained and that many of us have gotten under great difficulties through long years of toil and struggle.

Dr. Holliday, of Atlanta, said: The medical schools are taking steps demanding three years, and it shows they are not what they should be, or this would not be agitated by them. You can scarcely take up a medical journal that you do not see something about medical education. The second essayist made a point that I commend, and that is writing for the journals. How many of the profession in the South write for the journals every year? You come to these meetings; there may probably be half a dozen essays read, when there should be three times as many. That is because the members are inactive and do not take it upon themselves to write. I am engaged, as some of you know, in the publication of a journal; we need matter to publish sometimes and it is hard to get it. I write to gentlemen, and they say, "well, I will give you something sometimes," but it does not come. This thing of having meetings in sections is quite good.

Dr. G. W. Whitsett: I suppose I will stand all alone in my position, but it does seem to me like this three or four year's business is a piece of the biggest bosh. It is offering an insult to every young man that goes to college to say he has to go so many years until he can get a diploma. I think the colleges ought to raise their standards and say, "If you can get up to this in one year, you can have your diploma. If it takes you a dozen years, it will be so long before you get it." Don't let the Association say, "we will send young men to college and they must stay there three years;" and what is he doing there? He is trying to get the first rudiments of an education, while he might stay at home and get the same knowledge at one-third cost. I would like to see this society put itself on record as against that iron-clad rule that says a man has to go three years. I want to see the standard of education raised; have the colleges raise it, and then, if you can, go there and complete the course as soon as possible. You can go down to Chapel Hill and if you have only been there one year you can get a diploma, if you have completed the course.

Dr. S. P. Hilliard: I have a son that I am going to start out along that line, two years of office pupilage and then three years at college; that is worth a great deal.

Dr. G. W. Whitsett: But how many boys can you pick up in North Carolina that can give five years before they go to work?

Dr. Hilliard: They must have a good preliminary education.

Dr. Cryer: Dental education, of course has interested me very much. I am one of those unfortunates who did not receive the kind of education that our friend says we ought to have. I went from the plow handle out in Ohio; started the course at 34 years of age, and feel now that if I had had a college education, it would have been of very great benefit to me. I always deplore the lack of it. Dr. Morgan remarked that many of our best men came from the plow handle and not from the office. I believe that I have observed the same thing in the college that I am connected with. Dental education from my standpoint would commence by learning thoroughly the anatomy of the body with which you have to work. You cannot study anatomy except with the scalpel and cadaver. Take Leidy who, when a boy and before he went to college, studies anatomy by dissecting small animals, an example which it would be to the advantage of any intending student to follow. When a dental student cannot get the requisite knowledge from lectures given in his college, the hospitals and dissecting rooms of the medical schools are open to him at a slight cost There are also surgical and other operations in the mouth going on, that every dentist ought to understand and be familiar with, and the student has opportunities of seeing these performed without extra charge. Physiology can only be studied properly under experienced teachers, and with modern appliances. It is a pleasure now to be able to state that almost all dental colleges have dissecting rooms, physiological and chemical laboratories and a septic rooms where the gravest surgical operations can be performed and their treatment studied.

I went to a dental college knowing nothing of anatomy or dentistry; whatever knowledge I gained was obtained within the college. At that time, 1874-76, we had what was known as five year men, men who had practiced five years or more. They were allowed to graduate in one year. For some reason I am almost sorry we have not this class now, for I believe those five year men rendered valuable assistance to the young men by showing them how to put in practice the theories of the professors of mechanical and operative dentistry.

In this and other States I find that before a man can practice dentistry, legally, no matter where he has graduated, he has to come before your Dental Board of Examiners. I know one of the examiners in your State personally, and I know, too, that he has the ability to examine in his branch.

Now, if colleges turn out incompetent men, as no doubt they sometimes do, you have a custom-house officer, as it were, here, to say to the incompetent man, "you cannot practice in this State until you have better knowledge of your profession." This will tend to make colleges more careful of whom they send out to practice dentistry, and certainly the student will be spurred to greater diligence in his studies, looking forward to the fact that, even after obtaining his diploma, the aforesaid custom-house officer may debar him from practicing should he not be able to come up to the standard set down by the Board of Examiners of the State in which he desires to practice.

Dr. S. P. Hilliard: I want to make an explanation. I only meant to recommend what I said before; if a man go there and get through in a year, I would not prevent that.

Dr. J. S. Thompson, of Georgia, said: I have been waiting to hear what was said pro and con on this subject, and thought probably I might be needed in defending dental schools, but I think we are pretty much all on the same line. I do not think you have abused dental colleges. You must admit that they are making progress and doing all in their power to elevate the profession, but the profession should not expect too much from the dental school. A certain amount of preparation is necessary before entering a dental college, because there it would be impossible to give them a literary education. We received a communication a short time ago from one of the Boards—a resolution of the Board condemning colleges for passing young men that could not write sufficiently well for them to read their examination papers. Well, you must not expect dental colleges to teach young men how to write. You agree that there has been progress made in the education of the young men. The profession must do their full duty in the matter and look after them when they have graduated, and give them that assistance and maintenance which is necessary to develop the very best professional men. I congratulate these young men upon their papers, which were so well prepared. I enjoyed them very much.

On the subject of Dental Pathology and Therapeutics, Dr. Reminger read a paper prepared by Dr. W W. Rowe. In the discussion that followed, Dr. F. S. Harris said:

I should think that we might include in immediate root filling those cases where we expose the nerve in the operation. We are agreed that if there is any case which demands immediate root filling, it is a case when there is recent exposure, and then I, for one, am in favor of immediate root filling.

Dr. G. B. Patterson: I cannot agree as to the six weeks' treatment. I have treated many a tooth, but I have yet to treat one over two weeks, and I have been pretty successful. I find if two weeks won't do it, the forceps will. I do not believe in wearing anybody's patience out in treating six and eight weeks. I have had a few patients in my office that say they have had teeth treated for six and eight months and then they would have to have them out.

Dr J. E. Wyche: I do not think the essayist said anything about immediate root filling after a fistulous opening; with reference to the calculus being on the roots. I had a case some time ago that I would like to hear discussed. It was an upper molar, and on all three of the roots the tartar had gone almost to the apex. I want to know if anybody has had a case, and after getting the tartar off has put it back. I did not attempt it myself; I took the tooth out.

Dr. R. H. Jones: One thing connected with that subject in regard to pivoting teeth. I would like to ask if the gentlemen do not sometimes pivot teeth which have blind abscesses.

On the subject of Histology and Microscopy the chairman pleaded incompetency.

Dr. J. H. Durham: They say an honest confession is good for the soul, and I do plead incompetency to get up a paper on that subject that would interest men like these.

Dr. F. S. Harris: I was a party to putting up a job, so to speak, on our chairman. I wrote around to the members of the committee, and said, "let's be complimentary to our chairman and ask him to prepare that paper."

President C. L Alexander: It seems to me, you, gentlemen, have acted wisely. (Applause.)

Dr. Durham: I wish to thank you, sir, for your hearty approval of our course. (Laughter.)

No report on the subject of Dental Chemistry.

The subject of Dental Surgery postponed till day after to-morrow.

On the subject of Dental Appliances, Dr. Patterson said: Failing to obtain a paper, I thought I would tackle some one outside, and I wrote to our friend, Mr. Selby, who says he will have a paper a little later.

On the subject of Dental Hygiene, the paper of Dr. R, H. Jones was received with applause.

Dr. Reminger: Let me commend that paper. If we were to go around in this town we would find that they give attention to sewerage, and I suppose you have a health officer, whose business is to go around and see that the rubbish is cleared up. You may go in the country and even there you will observe great cleanliness in the homes of the people; but when you look inside of the mouth, you find a different condition of things, the sewerage is bad and the aspect anything but inviting. We ought to establish a toothbrush manufactory in Winston, and we ought to arrange it so that they can be sold cheaply, and they ought to be imposed on the people like newspaper men impose the newspapers on the people. If they won't clean up the rubbish get the legislature to pass a law to make the people clean their mouths. Sometimes it is an advantage to have catarrh a little, for when you approach the oral cavity of some people, it is not very inviting. We do need to tell the people something; beg the mothers to have the children clean their teeth, and I tell you it would be a good thing to have the mothers do it sometimes, too. Some people say, "I clean my teeth every morning when I get up. Then I want to laugh. Wash your hands just before you begin mixing mud! You need to brush your teeth after you have used them. Brush them after breakfast and more than all brush them after supper.

Dr. Hillard: There was a lady in my town whose children had bad teeth. She expended about $100 in having them put into good condition. She asked me, then, if something could not be done to save her two younger children's teeth. I told her that a tooth brush and floss silk would do it. She started at that time, when her boy was four and her girl was six years old; now the boy is fourteen and the girl sixteen, and I examined their teeth last week and there is not a speck of decay anywhere. If I had to depend on ladies who kept the advice of the dentists like she did, I would have to go to plowing or something else.

Dr. Wyche: I rise to add my hearty endorsement to Dr. Jones' paper, and I find it is one of the most difficult things to get mothers to make their children clean their teeth.

Mr. G. H. Patterson: I had a pleasant visit from the Doctor about a year ago, and I would like to ask him to go back again, if he can help one patient. I have one who never cleans his teeth, and to all my efforts he says that he is going to get a brush and do it. The structure is good but it has been literally eaten away by tartar.

Dr. Spurgeon: We should emphasize the point that the patients should clean their own teeth. A great many have the idea that about all the dentist is for, is to clean teeth. If you can get them to get hold of the idea that it would be just about as impolite in them to ask us to clean their ears or face, as to clean their teeth, I think a great many of them would clean them at home.

Dr. Conrad: I had proof of that. A very pretty young lady came into my office and asked me to clean her teeth, and I said, "why don't you ask me to wash your face? "Why," she said, "I always attend to that myself." "Well," I said, "if either has to be neglected, I want you to neglect your face and let me wash that, too." The young lady has not come back.

Dr. Thompson: It is a great pity that a paper like that cannot be brought before the public. If that could be done, it would be a very good thing; it is one of the most important preventatives of decay that we can suggest, if people would keep their teeth clean. Just think of it!

How many perfectly clean mouths do you see? It is a very rare thing for you to see what you would call a perfectly clean mouth. I have talked this to my patients over and over again, and I try to impress upon them the importance of keeping their teeth clean; I make them bring their brush and I show them how to clean their teeth, and I show them how by using a set of tin teeth, and I use every means to impress upon my patients the importance of cleaning their teeth, especially among younger patients.

At this point the session adjourned to meet the next day.

---

MORNING SESSION, MAY 26.

Call to order.

Roll call.

Minutes of last meeting read and approved.

Report of the Committee on Census report was as follows:

It was agreed to adopt a circular (changing the name, etc.) which was authorized to be sent by the Maryland Society to Congress showing that it is the desire of the profession in the United States to have this bill so amended that dentists will not be compelled to stultify themselves in answering questions of the census enumerators. And they simply want their representatives in Congress to see to it that this bill is so amended that dentistry shall be excepted from all tabulation just as the other professions are.

It was suggested that any member of the Association who knows a Congressman use his influence to secure the desired legislation.

Prosthetic dentistry. No report.

Dr. Turner spoke on the necessity of Chairmen of Committees making reports on the subjects under their charge, which was received with warm applause.

In answer to requests that he should speak on the subject, Dr. Turner said:

I appreciate very thoroughly the compliment that you have paid me, but if you will pardon me, to come down seriously to the discussion of this subject, I trust you will forgive the earnestness with which I have expressed myself, and I, of course, feel perfectly good-natured about the whole thing.

In regard to Prosthetic Dentistry, I scarcely know how to commence the discussion, except to say that I believe that prothetic dentistry means the application of artificial teeth. Now then, if the application of artificial teeth to the mouth is the true definition of the term, then I would like to suggest that this matter of bridge-work, which seems to have occupied the attention of a great many gentlemen of skill and ability, should be brought out. I have not had very much experience in that line, comparatively speaking, but there are many points yet to be learned about bridge-work. You will hear one man who says: "I am very thoroughly convinced that this is a step in the right direction;" and another man who says, "I believe you have to go very slow with bridge-work." There are many points which would be of interest to this Association upon this subject, and I feel that there are gentlemen present who would probably enlighten us upon the main points involved. There is the question as to whether the bridge should rest upon the gum, or whether it should be elevated or removed, separated from the gum, leaving a space between. Now, I saw a member of the dental profession in Atlanta putting in a bridge and he put his plate and fastened it right down on the gum and he contended that was the proper course. I differ with this gentleman.

Then another question as to what effect the ordinary band has upon the gum. It is frequently the case that where a bridge has been put in for months, and the gum is apparently in a normal condition, when you come to examine you will find a piece of bridge work where the execution has been apparently perfect, and still you will find conditions which threaten to be so serious that decay will set in. My idea is that a bridge, where there are any number of teeth, ought not to rest directly upon the gum; there ought to be sufficient space to remove food as expeditiously as possible, and I feel that where I have tried to put a bridge down very closely, I have found more or less trouble, because the average patient is not very careful sometimes. I do not claim to have done very much in this line, and I am interested upon the subject, and I would like some gentlemen who understand these things to say something to this Association.

Dr. Morgan: I am willing to give you the benefit of what little experience I have had in bridge work, and more particularly from observation. I have been interested in the subject since the year 1879 when I saw the first all-gold crown that I ever saw. Like Dr. Turner, I disagree with those who follow the practice of adjusting bridges that rest upon the gum, or making what is sometimes called the saddle bridge. Saddle bridges, or bridges that are adjusted right over the molars or bi-cuspids or cuspids, it is impossible to keep clean; I believe it was Prof. Taft who said that he never got within three feet of a person wearing them that he could not detect the fact from the odor of the breath. I do not, in my own practice, undertake to make a bridge where I have not two abutments. I must have a lodgment at each end. The day of the insertion of ten, twelve and fourteen teeth upon three, four or five teeth is past and gone and those bridges are now put in in sections.

None of us will deny that we have yet determined no fixed set of rules by which we may adjust these bridges. A bridge that has more than one tooth beyond its point of anchorage, is in my opinion a very risky piece of work. We all have our peculiar modes, some having very much shorter cuts than others; some doing the work as we saw here yesterday at one sitting, while others arrange their points of anchorage and afterwards adjust it on a model, having the piece finished up for insertion in the mouth after its completion. We had shown us at Morehead City last summer what was termed by Dr. Marshall the "anchor plate." Since then I have interested myself somewhat in these anchor plates, and as it seems, from those with whom I have talked since being away, that they are not very generally used or understood by us, that might be of more interest to you than a discussion of the bridge.

The anchorage plate is a plate that is made removable. Where there are one or two teeth remaining in the mouth we change our parallel and cap with crown; after that a band of gold is telescoped over the teeth and the plate is anchored to these telescopes, so that when the plate is removed from the mouth, or when the plate is inserted, the point of bearing is not upon the crown tooth so much as it is upon the jaw. Possibly the citation of a single case would be of more value than more dealing generalities.

A case presented itself at the office of a gentleman whose anterior teeth below were all that remained except the second bicuspid on the right side. Those anterior teeth were extracted, the first bicuspid having been lost some time previous; the second bicuspid on the right with the cuspid on the left were gone. An impression was taken after having the crown in position. From this impression a model was made; bands were made to adjust themselves to the crowns, and the plate carried into the mouth and the bands telescoped over the crowns. An impression was then taken with the plate and bands in the mouth. A model of fire clay was run. Upon the anterior portion six teeth were placed by soldering. This was better because of the solidity that it gave to the plate. The posterior teeth—the molars—were put on with rubber, and Gilbert's Granular gum was put on. It was then, when finished up, ready for insertion, and I am satisfied that nothing that could have been done for this gentleman could have been so serviceable. The resting is perfectly firm, and in some instances the teeth that were comparatively loose under this treatment become quite firm and steady, even if they had been previously affected with Pyorrhœa Alveolaris. It seems to be the case in my practice that teeth which have been suffering with Pyorrhœa Alveolaris and which have been capped with the gold crown, which I regard as the greatest contribution to dentistry since the war, that teeth that have been loose become firm, and that frequently if the cap has been thoroughly and properly adjusted with due skill, so that there is no impingement of the gum, and the most of those with whom I have talked say that they can better adjust those that are manufactured than those that they make themselves. These teeth, I say, that are quite loose will become quite firm and steady in their positions. In the manufacture of these plates all pressure is relieved from the gum. All of you who have had an opportunity of observing the old fashioned clasps remember how the clasp just at the neck of the tooth irritated the gum. In making these anchor plates, we get rid entirely of that difficulty and in those that I have had an opportunity of observing, that have been in the mouth for six or eight months, the gum is perfectly healty. (Applause.)

Dr. Smith: I do not feel like the Irishman who said he did not hear both sides of the question because it always confused him. I would like to hear both sides of the question. So far as I am concerned, I regard bridge work as a great boon. But I have never seen a saddle bridge put in, that had been worn any length of time, which has not given rise to inflammation and with serious consequences following. I have met those who said it was impossible to make bridge work in the laboratory, that it must be made in the chair. I do not make my bridge work at the chair. A great deal depends on getting a perfect impression. I force the modeling composition around the tooth and let it stay there until it gets partially hard. After it gets partially cold I take a knife and cut out the modeling composition all around the tooth, take a little modeling composition that is very soft, run it through the flame of the lamp until it is very hot, drop this little ball of modeling composition into the impression, then replace it, and I get a most perfect impression—it is absolutely correct and it does not take it long to cool off. I take that out and send it to the laboratory and have a model run. The model is returned to me and I take my knife and cut about the teeth, cutting down a little, and after having examined the teeth I prepare that model and send it back to the laboratory, and my young man in the laboratory takes tin or lead foil, makes a band to go around that tooth allowing it to extend where I have cut away under the gum. He takes this pattern and cuts the gold; with a little wire he wraps it; putting it in the flame solders it, so it comes back to me as a band. Sometimes I find that I have to send it back and cut it, but in the majority of cases the band fits the tooth. I mark where I want it cut down, and send it back to the laboratory, where he has swaged a cusp for it. This he solders and sends it to me again very soft. I put it into the mouth and let the patient bite into it, so a perfect occlusion is made. Then I send it back again and he fills it up with solder, then he sends it to me again and I take it out in a plaster impression. Then the teeth are adjusted, first trying the color, and then the bridge is ready to put in.

I do not approve of bridge work in all cases, gentlemen. There has been so much harm done in bridge work. It is not the fault of bridge work but the fault of some men. Do not undertake to put bridge work into the mouth unless you are certain that it will be good for a number of years. I have seen bridges put in that after five or six months failed and came to pieces.

Another thing, try and have constant communication between your patients and yourselves. My patients are pledged to come to see me every six months, in some cases every three months. I would not put in a bridge for a patient who would not promise to consult some dentist every six months. (Applause.)

Dr. E. L. Hunter: I fear that some of the gentlemen think I am opposed to bridge work entirely, for the simple reason that I have condemned its abuse. I just wanted to say that because I feared I stood before the Society as condemning bridge work.

Dr. Cryer: It is one of the best processes, this capping the teeth with gold crowns which become anchorages to hold plates. Dr. Morgan spoke of the tooth becoming firmer. I think it is perhaps for this reason: A tooth standing alone is like a man standing alone—he has nothing to lean against. By putting a plate in by this process it is held in position, and at the same time the crowned tooth holds the plate in position. (At this point Dr. Cryer spoke of a case before the Society—that of the elderly gentleman who had the cancer removed from his nose. The gentleman came forward and Dr. Cryer explained to the Society the details of the process of making the partially artificial nose.)

Dr. Thompson: I want to add my testimony to all that Dr. Morgan has said with regard to the anchor plate, and in addition I would like to suggest making metal models. This (showing model) is the model of a case I made in practice. Of course in bridge work you could not, at least I do not recommend using those extension bridges and using the saddle. I do not recommend bridge work where you do not have two abutments, except that I differ from Dr. Morgan in that respect that I frequently insert front teeth, one or possibly two teeth on one root. In this case I crowned the first bicuspid on the left and the second bicuspid on the right with gold, made a telescoping band to fit over them, and the great advantage you have here by a metal model, you can get that direct from the impression. Your telescoping band you can fit to these crowns and then attach your plate to this band and you have no breaking down; in pressing up your plate your model holds its shape and you get a more accurate fit, I think, and your bands are held better in position. I have been using, since Dr. Marshall recommended it at Morehead City, this process very extensively, and it has taken the place of bridge work in my establishment; it is nothing more than removable bridge work. My experience has been as with Dr. Morgan; where you have teeth with a double duty placed upon them, nature comes to their assistance and they become firm; I have yet to see a single tooth that I have not thought was improved by this attachment.

As to Dr. Smith's process of making bridge work, I do not agree with him in making bridge work in the laboratory; I can not make an accurate fit of the neck of tooth except in the mouth. In the first place, I can not get an impression of the part that I wish to hook closely if my band is beneath the gums. In preparing my crowns I cut it down in a conical shape, and I contend that you can not get an accurate impression of the neck of the tooth beneath the gums. After I get my teeth all ready for receiving the crown, I can fit it while I am getting an impression of it; I put my crowns direct to the teeth. In making a bridge, I have an original and quick method. For instance, if I were going to bridge from this second bicuspid to the third molar, I would prepare those crowns and prepare the teeth for receiving the crowns and crown them; I use nothing but gold crowns—I have abandoned the porcelain-faced crowns; I then stamp up the gold crowns; those crowns are then trimmed so that they do not touch the gum. I do all this while the patient is sitting in the chair. I place the crown which I have stamped up in position in contact with the crown upon the natural teeth, the patient closes down and presses the wax down, holding it in position; then I mark where this crown comes in contact with the crown upon the teeth so that I can replace it after taking it from the mouth. I remove that crown and the crown from the natural teeth, and with my plyers I hold them together and drop a little piece of solder just between them which attaches them together. That model crown is attached to the crown which goes upon the natural teeth that is replaced in the mouth; if the crown stand up a little, the teeth come right down and press it into place. All these crowns I use, in making. pure gold; it is very soft indeed; if you have got it a little too high, the teeth will put it in place; if you have got it too low, you can run underneath and raise it; then, by carefully removing the whole skeleton, you solder it. These model crowns are filled in solidly; I do not pretend to try to leave a vacuum there, but I fill them, using for this purpose the old filings, covering them over with the solder, and you can see how quickly you can make a bridge of that sort—you can do it at one sitting. While I have the patient in the chair I can stamp these crowns up in two minutes, and. if you wish, by keeping the patient waiting a short time I can have the bridge ready to go into the mouth; you can get it up as rapidly as you please, and you can construct a bridge of this sort more quickly than any thing that I have ever seen. It is solid metal all the way through, and I have bridges of that sort that are being worn from the cuspid back to the third molars, and they are perfectly strong and satisfactory, never having had but one to give way, and that was one of the first that I ever constructed. This is my process of making bridges.

Now, in making bridges of that sort, I always leave a space between the gum and the bridge, so as to give plenty of room for keeping it thoroughly cleansed.

I wanted to say something on the subject of metal models. I have used almost exclusively metal models; some of you saw yesterday how I prepared the impression. After the impression in plaster is taken, I have a little saw that I made myself out of a piece of clock spring, with which I saw through the impression an air-gate allowing the air to pass out. In operating in metal. as it comes together, unless you have extracted the air, a little portion of air is collected under there, and you have a defect in the model. By passing this little saw through you have plenty of escape for the air, and the metal will give you a perfect model. These models are very easily made. After you have got the impression and sawed little air-gates through it, you take a piece of tin and just surround your impression; that tin-foil is wrapped right around the outside of your impression; that is the only preparation you have to make of the impression. I do not use marble dust with my impression; this impression is surrounded by plaster dried out thoroughly, then you pour block-tin into the impression after it is dried, and you get a perfect model of it. As you know, there is a slight contraction of the tin and an expansion of the plaster, but the contraction of the tin is more than the expansion of the plaster. You have a metal then that you can work upon without any danger of breaking down; your plate comes out polished—it can be trimmed up. And another advantage of the metal model: after you have placed it upon the articulator, you go to work and invest it, press up your plate, and then you can replace it upon the articulator and see that you have gotten your articulation correct; you place thls back upon the articulator, and, by bringing the articulator together, you can see exactly where it needs grinding. These are a few of the advantages of metal models.

Another thing I have said, wherever I go: the suction plate covering the roof of the mouth is one step in the right direction. We have no idea what a great inconvenience it is to have the roof of the mouth covered; it was intended by nature to be left uncovered, and we must avoid covering wherever we can, I am using these anchor plates wherever I can, and I find with the metal models you can make an accurate fit. (Applause.)

Dr. Smith: I do not propose to be misrepresented by my friend Thompson, here; I did not say, and I do not wish to be put on record as saying that I did not fit my bands in the mouth. I said that I did not *make* the bands in the mouth. If it is possible to get a correct impression of a tooth, it is certainly much more easy to get a perfect adaptation of a sheet of gold to that tooth in the laboratory on the model than it is in the mouth, where you are embarassed by saliva and the teeth and lips of the patient. If Dr. Thompson will pursue my method, he can get a perfect impression.

At the conclusion of the discussion between Drs. Smith and Thompson, Dr. Holliday said:

I have had very little experience with bridge work. I have done considerable crown work, but not much bridge work; and, after the ground has been gone over so thoroughly by Dr. Morgan and Dr. Thompson, I do not know that I have anything to add that would be new or different from what they said. I agree with the gentlemen that have gone before, in the adjustment of the crown. I pursue Dr. Thompson's method, somewhat; I think that I can do it better in that way. An impression can be made, and a perfect impression of the root, too, even under the margin of the gum. In preparing the root, it necessarily presses the gum out of the way, and the modeling composition will force itself under the margin of the gum.

After repeated calls, Dr. Alexander said:

I have really nothing to say. All the crowns I make, I make them with the patient in the chair. I do not use models or take an impression.—*Southern Dent. Jonrnal.*

[To be Continued.]

